# Mobile HIV Testing Through Social Networking Platforms: Comparative Study

**DOI:** 10.2196/25031

**Published:** 2021-03-26

**Authors:** Piao-Yi Chiou, Nai-Ying Ko, Chien-Yu Chien

**Affiliations:** 1 Department of Nursing National Taiwan University Hospital Taipei City Taiwan; 2 School of Nursing College of Medicine, National Taiwan University Taipei City Taiwan; 3 Department of Nursing College of Medicine, National Cheng Kung Tainan Taiwan

**Keywords:** HIV testing, internet-based intervention, men who have sex with men, mobile apps, mobile phone, risk-taking, social networking, voluntary counseling and testing

## Abstract

**Background:**

Improving HIV screening in key populations is a crucial strategy to achieve the goal of eliminating AIDS in 2030. Social networking platforms can be used to recruit high risk-taking men who have sex with men (MSM) to promote the delivery of voluntary counseling and testing (VCT) as mobile HIV testing. Therefore, client recruitment and availability of mobile HIV testing through social networking platforms requires further evaluation.

**Objective:**

The aim of this study is to compare the effects of targeting high risk-taking MSM and HIV case finding between two mobile HIV testing recruitment approaches: through the traditional website-based approach and through social networking platforms.

**Methods:**

A comparative study design and propensity score matching was applied. The traditional VCT model, that is, the control group, recruited MSM through a website, and a trained research assistant visited the walk-in testing station at a gay village on Friday and Saturday nights. The social networking VCT model, the experimental group, recruited MSM from social networking platforms by periodically reloading into and conducting web-based discussions on dating apps and Facebook. The participants then referred to others in their social networks via a popular messenger app in Taiwan. The test was conducted at a designated time and place during weekdays by a trained research assistant. Across both modes of contact, before the mobile HIV testing, participants needed to provide demographic characteristics and respond to a questionnaire about HIV risk-taking behaviors.

**Results:**

We recruited 831 MSM over 6 months, with a completion rate of 8.56% (616/7200) in the traditional VCT model and 20.71% (215/1038) in the social networking VCT model. After propensity score matching, there were 215 MSM in each group (mean age 29.97, SD 7.61 years). The social networking model was more likely to reach MSM with HIV risk-taking behaviors, that is, those seeking sexual activity through social media, having multiple sexual partners and unprotected anal intercourse, having experience of recreational drug use, and never having or not regularly having an HIV test, compared with the traditional model. HIV positive rates (incidence rate ratio 3.40, 95% CI 1.089-10.584; *P*=.03) and clinic referral rates (incidence rate ratio 0.03, 95% CI 0.001-0.585; *P*=.006) were significantly higher among those in the social networking VCT model than in the traditional VCT model.

**Conclusions:**

Through effective recruitment strategies on social networking platforms, the social networking VCT mode can be smoothly promoted, as compared with the traditional VCT model, to target high risk-taking MSM and increase testing outcomes.

## Introduction

### Background

Improving HIV screening in key populations is a vital strategy to reach the goal of 95-95-95 and eliminating AIDS in 2030 [1]. A study estimated that 88% of people with HIV in Taiwan were aware of their infection status in 2019 [2]. In addition to the current screening model, a more effective testing approach should be applied, such that 12% of the undiagnosed targets also undergo HIV testing.

Men who have sex with men (MSM) who had demonstrated unsafe sexual behaviors constitute a major group with HIV infection in Taiwan and globally [3,4]. To improve the uptake of voluntary counseling and testing (VCT) of MSM, mobile HIV testing, which emphasizes on the use of vehicles to deliver VCT into the community, was developed [5,6]. Mobile HIV testing employs VCT outside hospitals to target different populations and hard-to-reach groups [7-10]. To improve the HIV case findings, mobile HIV testing information should be effectively disseminated.

Usually, the client awareness of mobile HIV testing mainly results from its announcement on several platforms such as websites, radio, flyers, posters, and notifications by advocates [7-10]. Due to the high availability of smartphones, social network platforms that include mobile geosocial network apps (GSN apps) with GPS (eg, Grindr, Jack’d, and Hornet) and web-based communities (eg, Facebook [FB]) have gained a large number of web-based users and have become a popular avenue for MSM to search for health information and meet sexual partners [11-14]. High risk-taking behaviors for HIV infection, such as having multiple sexual partners, low frequency of condom use, and a high percentage of recreational drug use, have increased by using social networking platforms [15-18]. Therefore, the use of social networking platforms may also help gather mobile HIV testing information and encourage VCT among high risk-taking MSM [19,20].

In this research, HIV testing-eliciting messages and web-based discussions over instant messaging through social networking platforms were used to encourage the use of HIV VCT and self-testing among MSM [21-25]. However, there is an urgent need for more effective recruitment measures with tailored operations of social networking platforms among high risk-taking MSM. MSM users on social networking platforms could change depending on the time and place of the user who logs in. Active strategies, such as periodically reloading into GSN apps at different times and locations and setting up a fun page on popular web-based communities, could increase opportunities for web-based users to expand the information exposure of mobile HIV testing. The inability to coordinate time and location is one of the major barriers for MSM to reach the VCT [26,27]. Providing more flexible and individual HIV screening times and locations for MSM users could facilitate the accessibility of mobile HIV testing.

In summary, social networking platforms could be a direct path to target high risk-taking MSM. It is necessary to promote mobile HIV testing via social networking platforms, to deliver VCT according to the assigned time and location by MSM, and to verify the effects of HIV case finding outcomes.

### Objective

The objective of this research is to compare the effects of targeting high risk-taking MSM and HIV case finding between 2 mobile HIV testing recruitment approaches: through the website and through social networking platforms.

## Methods

### Study Design

A comparative study design was applied, wherein two VCT models were evaluated, and purposive sampling was employed. The traditional VCT model is the most popular model in Taiwan and was therefore chosen as the control group. Specifically, VCT would be applied at a certain period of time at a testing station in the community; this implementation was announced through a website. In contrast, the social networking VCT model included scheduling through social networking platforms and providing VCT at designated times and places. The most popular social networking platforms, namely, Grindr, Hornet, Jack’d, FB, and Line (a popular mobile instant messaging app in Taiwan), were selected based on the previous year’s data (MOHW106-CDC-C-114-000115). The study covered the period from May 1, 2018, to November 1, 2018, in Taipei and New Taipei City. These two cities were chosen because of their higher prevalence rates of HIV infection in Taiwan.

### Participants

Inclusion criteria were self-reporting as MSM, having sexual experience, being older than 20 years, and being literate. Exclusion criteria were those who self-reported as not MSM, not having any sexual experience, and previously known to be HIV positive.

### Ethical Considerations

The study was approved by the institutional review board of a medical center (18MMHISO25e). All the participants’ data were processed anonymously using codes. During the HIV confirmation test process, the participants did not need to disclose any personally identifiable information to the research team to maintain their privacy. After completing the consultation, questionnaire, and testing, each participant was eligible to receive a gift worth US $3.27.

### Procedure and Data Collection

#### Traditional VCT Model

The testing information of the traditional VCT model included free and anonymous HIV rapid testing; the testing time and location were announced every day during the study period via the public website of a municipal hospital. The traditional VCT model was conveyed to a screening station located at the entrance of a gay village between 6 PM and 10 PM every Friday and Saturday in Taipei City. The participants could actively browse the website, pass and see the outreach station, or be referred by their networks to learn about the testing information and to access the VCT. Moreover, the traditional VCT model provides walk-in services without an appointment.

A trained research assistant conducted pretest counseling and explained the research design and purpose. After obtaining written informed consent, the participants were asked to complete a questionnaire, and the pre- and posttest counseling of the rapid HIV test was performed for each participant. The participants were informed of the test results immediately after the test. For the participants with a positive test result, the research assistant accompanied them to the clinics for further confirmation via a diagnostic test. For the participants with a negative result, crucial resources about HIV prevention, such as pre-exposure prophylaxis (PrEP) and postexposure prophylaxis (PEP), were introduced, and a referral was made for them.

#### Social Networking VCT Model

The social networking VCT model recruited MSM from social networking platforms and provided a free and anonymous rapid HIV test at a time and place designated by the participants.

**Figure 1:**
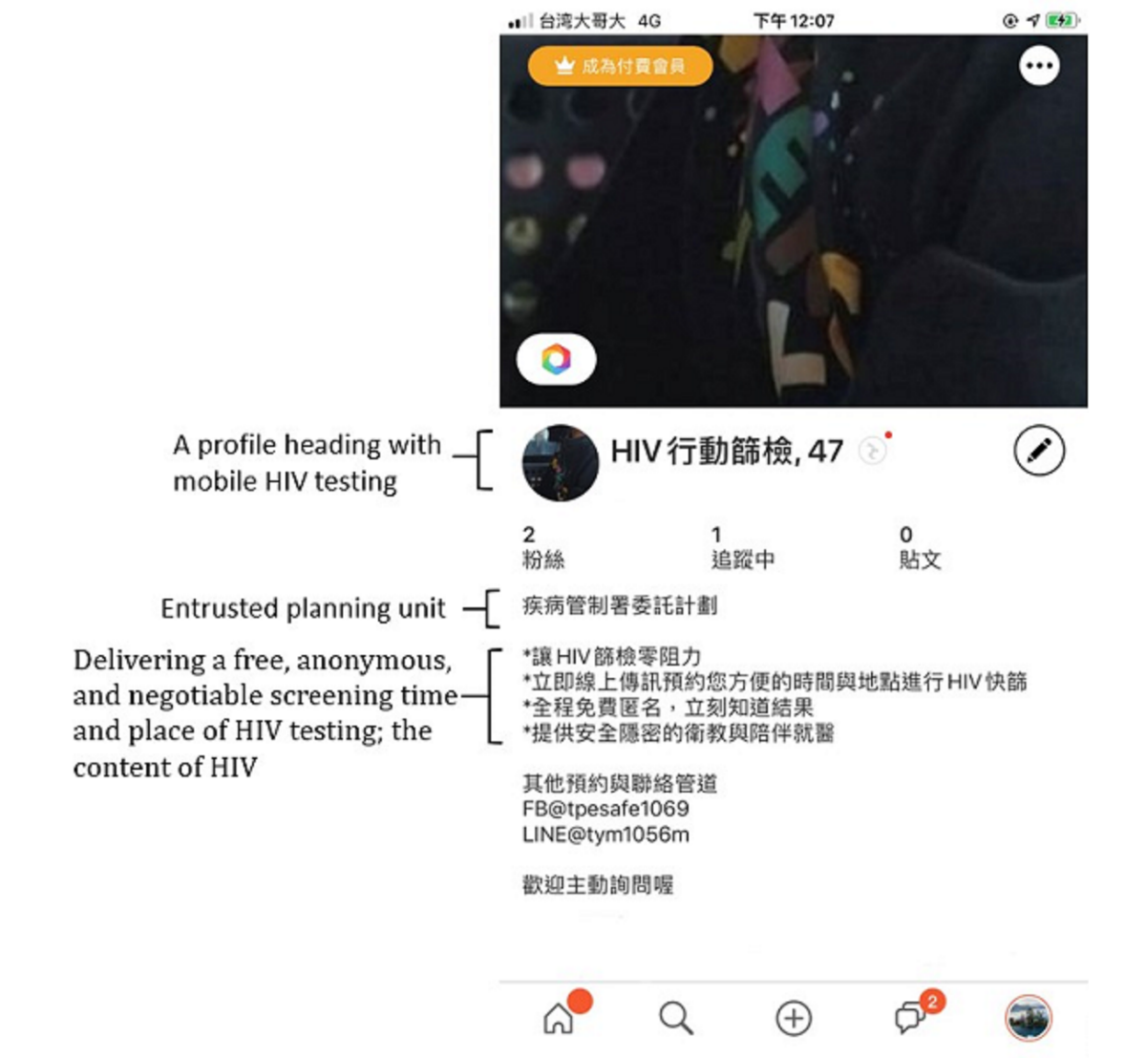
The profile of the mobile HIV testing in geosocial network apps.

A profile heading with *mobile HIV testing* and a picture of a well-trained research assistant was set up (Figure 1) in the GSN apps: Grindr, Hornet, and Jack’d. The profile clearly introduced the program’s purpose; the name of the entrusted planning unit; reservation for delivering a free, anonymous, and negotiable screening time and place; and the content of HIV testing services to enhance the audience’s trust [28]. The users appear on a grid with 3 to 4 profile photos in each column in Grindr, Hornet, and Jack’d, who were within a 7-mile radius from the research assistant. The main log-in location of the research assistant was the program institution at Shipai Road, Beitou District, Taipei City. The other log-in locations changed according to the designated testing locations of the participants in Taipei and New Taipei City. Two strategies of client mobilization and recruitment were applied to the GSN apps [20,29,30]: (1) reloading once every 2 hours, a total of 4 times per day, from 10 AM to noon and 1:30 PM to 7:30 PM, on weekdays, onto each app to display our heading and profile, and allow nearby web-based users who were interested in mobile HIV testing to actively tap or send a private message to the research assistant, and (2) the research assistant provided one-on-one web-based discussions by using standard contents, including the research purpose, the privacy and rights of the participants, risk-taking behavior, and the window period of HIV infection. Individual mobile HIV testing appointments were decided after discussion.

**Figure 2:**
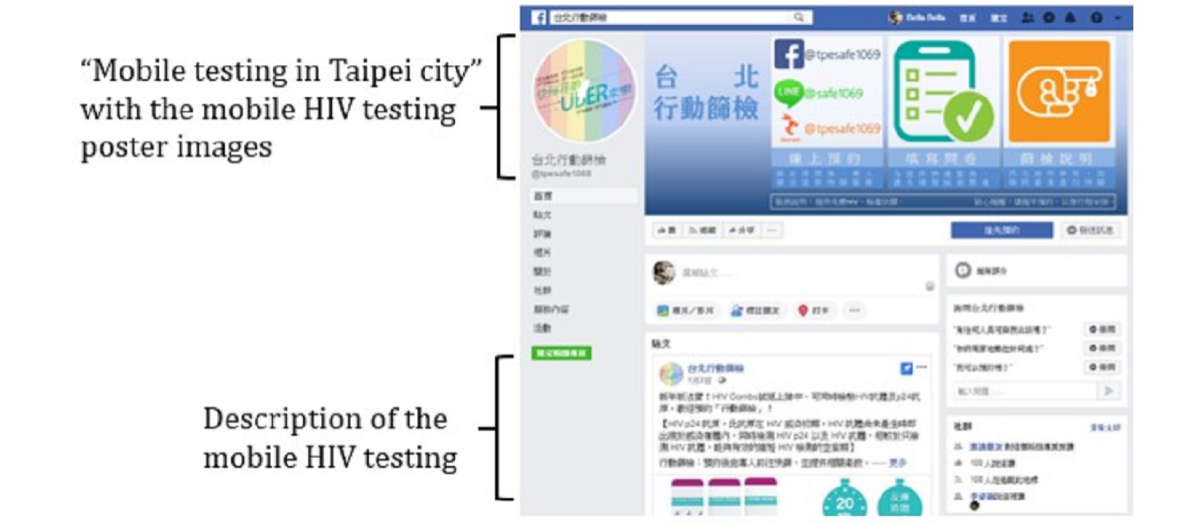
The profile of the mobile HIV testing on the Facebook page.

An FB fun page (Figure 2) was created with *mobile testing in Taipei City* under the top section of the page with the mobile HIV testing poster images, and a clear description of the mobile HIV testing was provided to raise awareness and build rapport with the users on FB. Potential participants on FB who were interested in mobile HIV testing could send a private message to have a one-on-one discussion and make an appointment with the research assistant using standard content. A previous study used a similar recruitment method using FB [31].

We encouraged the participants who had finished the mobile HIV testing to refer to their social network to make a mobile HIV testing appointment through a quick response code for a messenger app (Line app).

The same research assistant delivered the VCT according to the appointment at a designated time between 10 AM to noon and 1:30 PM to 7:30 PM on weekdays and at a convenient place. The ensuing test counseling, testing procedures, and posttest services were the same as in the traditional VCT model.

Table 1 shows the content comparison of the 2 VCT models.

**Table 1:** The content comparison of the 2 voluntary counseling and testing models.

Process	Traditional VCT^a^ model	Social networking VCT model	
The client mobilization and recruitment strategies	The screening information is announced through a public website of a municipal hospital every day during the research period Subjects pass and see the outreach station Referral by their networks	In GSN^b^ apps: (1) reloading the user location information in the apps, namely, Grindr, Hornet, and Jack’d, once every 2 hours, a total of 4 times a day during 10 AM to noon and 1:30 PM to 7:30 PM on weekdays to display our mobile HIV testing heading and profile for nearby web-based users, and (2) providing one-on-one web-based discussions Create a Facebook fun page and an ID in a messenger app (Line app) to provide the one-on-one private message discussion Encourage the participants who had finished the mobile HIV testing to refer their social network through the Line app	
Time of VCT	6 PM to 10 PM every Friday and Saturday	Designated by the participants during 10 AM to noon and 1:30 PM to 7:30 PM every weekday	
Location of VCT	An outreach screening station at an entrance of the largest gay village in Taipei City	Designated by the participants at a convenient place in Taipei and New Taipei City	
Testing appointment	No need to make an appointment	Need to make an appointment	
Testing method	Rapid HIV test		Rapid HIV test
Staff	A trained research assistant		A trained research assistant
Result notification	Informed of the HIV testing result immediately after the test		Informed of the HIV testing result immediately after the test
Posttest services	The referring of confirm test and PEP^c^ and PrEP^d^ can be arranged directly according to the testing result		The referring of confirm test and PEP and PrEP can be arranged directly according to the testing result

^a^VCT: voluntary counseling and testing.

^b^GSN: geosocial network.

^c^PEP: postexposure prophylaxis.

^d^PrEP: pre-exposure prophylaxis.

### Instrument

#### Demographic Characteristics Questionnaire

Participants provided demographic information, such as age, education, employment status, religion, and whether coming out of sexual orientation.

#### HIV Risk-Taking Behaviors Questionnaire

HIV risk-taking behaviors included days since the last unsafe sex, seeking sexual activity through social media, the nature of the relationship with the current sexual partner within the past 3 months, the frequency of anal intercourse and of condom use during anal intercourse within the past 3 months, recreational drug use, history of sexually transmitted diseases, having had a prior HIV test, regularity of this testing within the previous year, and previous PEP and PrEP.

#### Rapid HIV Test

DETERMINE HIV–1 and 2, antigen and antibody combo was used as the rapid screening tool in this study, which was licensed by the Taiwan Food and Drug Administration with a high sensitivity of 100% and a high specificity of 99.5% [32]. It takes only about 15 min to obtain the results, and it can be performed by trained staff.

#### Propensity Score Matching and Multivariable Statistical Analyses

As the subjects who accepted the anonymous HIV testing could not be randomly assigned, propensity score matching (PSM) was applied using SPSS (SPSS Inc.) software to perform the matching in a 1:1 ratio with a caliper distance of 0.001 between the 2 VCT models to control the extraneous variables and improve the homogeneity of the characteristics of the participants in the 2 VCT models. Perfect PSM was based on age, education, and employment level [33]. The comparison of demographic data and risk-taking behavior (secondary outcome) between the 2 models was analyzed using a paired *t* test for continuous variables and a chi-square test for categorical variables. Percentages were used to describe the distribution of the time and location of the social networking VCT model. All testing locations were imported into Google Maps to calculate the coverage area of the social networking VCT model. The incidence rate ratio (IRR) and 95% CI were used to test the significance of the primary outcome of HIV testing results and the referral rates after a positive result.

#### Estimation of Sample Size

G*Power was used to estimate the matched pair sample size. On the basis of an effect size of 0.25 and power of 0.95, the sample size in each group was calculated to be 175. At a loss rate of 20%, the sample size was calculated to be at least 210 for each group.

## Results

### The Recruitment and Completion of 2 VCT Models

**Figure 3:**
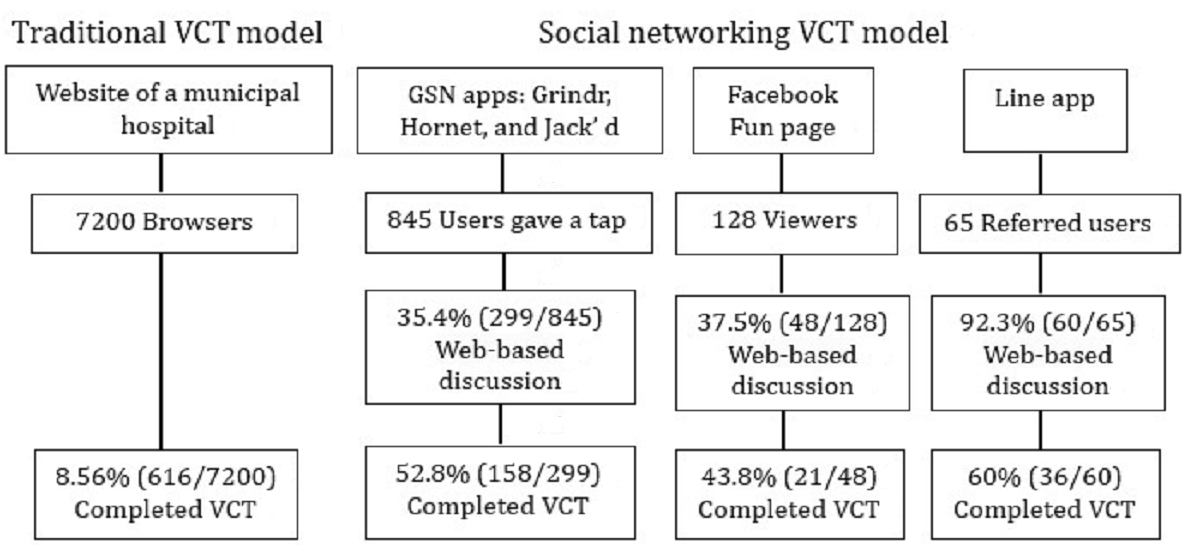
Attrition diagram for the 2 voluntary counseling and testing models. GSN: geosocial network; VCT: voluntary counseling and testing.

Figure 3 shows the attrition diagrams of the 2 VCT models. In total, 831 MSM participants were recruited into the 2 VCT models during the research period: 215 in the social networking VCT model and 616 in the traditional VCT model. An estimated 7200 visits, including website browsing, passing, or referrals for obtaining the VCT in the outreach station, achieved a completion rate of 8.56% (616/7200) in the traditional VCT model. Of the 215 participants in the social networking VCT model, 73.5% (158) were recruited via GSN apps, 16.7% (36) were recruited via the Line app, and 9.8% (21) were recruited via the FB fun page. A total of 845 GSN app users in Grindr, Hornet, and Jack’d actively gave us a tap, of which 35.4% (299) entered a one-on-one web-based discussion with the research assistant and 52.8% (158/299) received the VCT. After 48 private message discussions from 128 viewers through FB fun page, 44% (21/48) users received the VCT. There were 65 referred users sending the message to as through Line app, of which 92% (60) of them had one-on-one web-based discussion and 60% (36/60) of those received the VCT. The overall completion rate of the social networking VCT model, which was calculated through the number of the participants who have completed the mobile HIV testing (n=215) divided by the sum of number of the subjects who giving as the tap in GSN app (n=845), being viewers in the FB fun page (n=128), and being referred through the Line app (n=65), the result is 20.71% (215/1038). The main reasons for not accepting social networking VCT include having no response message, having been screened recently, having no risk-taking behavior, and not wanting to know the result.

**Figure 4:**
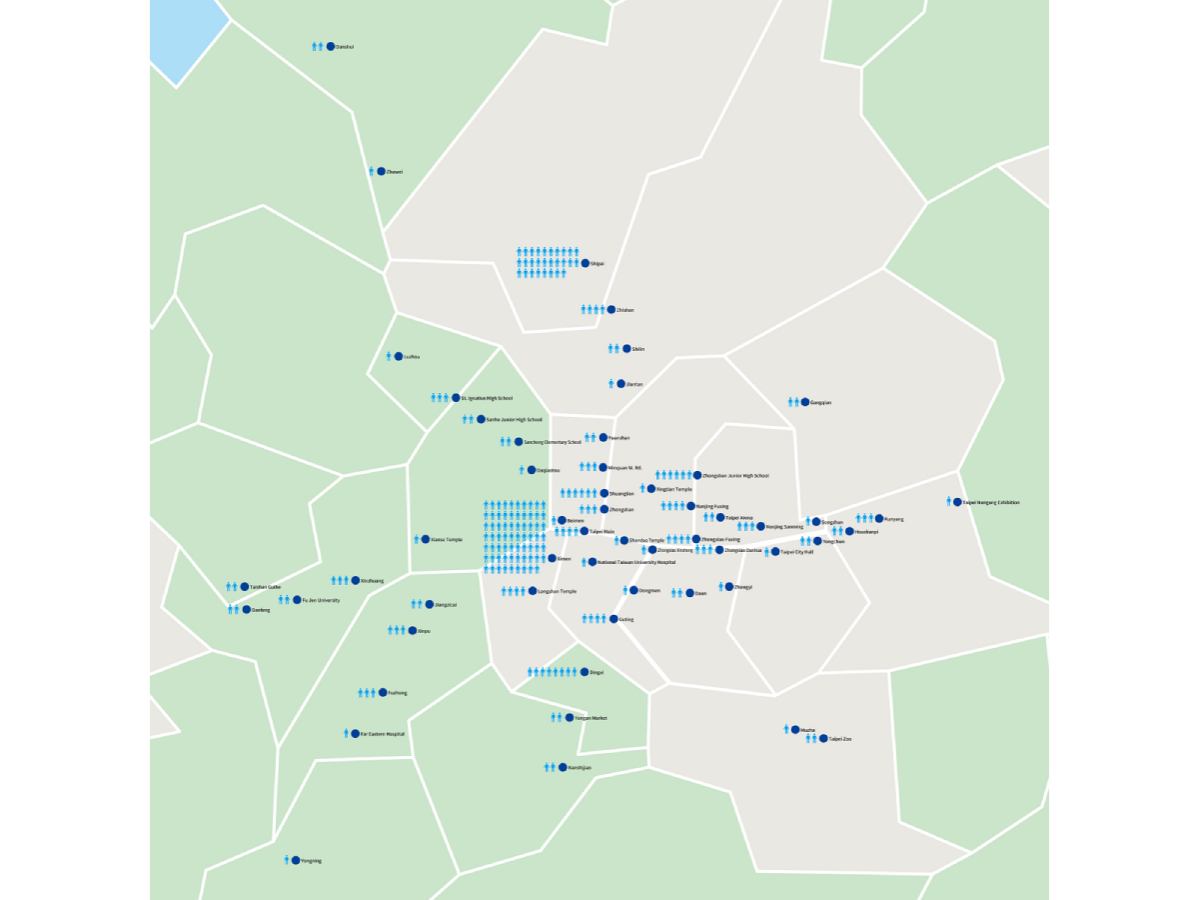
The distribution of tested men who have sex with men in social networking voluntary counseling and testing model in Taipei and New Taipei City.

The most popular designated testing period of the social networking VCT model was 1:30 PM to 5:30 PM (129/215, 60.0%), followed by 10 AM to noon (48/215, 22.3%) and 5:30 PM to 7:30 PM (38/215, 17.7%). The testing locations of the social networking VCT model and reloading locations of GSN apps by mobile phones expanded to adjacent walking areas within the 55 Taipei mass rapid transportation (MRT) stations (Figure 4). The total area of the social networking VCT model was calculated after connecting the farthest MRT station and was estimated to be 235.28 km², which covered 10.12% of the area of Taipei and New Taipei City. Figure 4 also shows the distribution of screening numbers for MSM. There were 29.3% (63/215) participants who requested mobile HIV testing of the social networking VCT model at their own house, 27.0% (58/215) at a convenience store such as 7-Eleven or Family Mart, and 24.2% (52/215) at a fast-food restaurant or café such as McDonalds or Starbucks. There were 14.0% (30/215) participants who requested mobile HIV testing in an outdoor area, such as a street side, a garden, or a school campus corner, and 5.6% (12/215) at a gay bar.

### The Comparison of the Characteristics of MSM Between the 2 VCT Models

After PSM, 215 participants with a perfect match score were selected for each model. Table 2 presents a comparison of the participants’ characteristics between the 2 models. The mean age of all participants was 29.97 years (SD 7.61); most had college- or university-level education (294/430, 68.4%), were employed (296/430, 68.8%), and did not practice any religion (224/430, 52.1%). Approximately 60.5% (260/430) of the participants responded that they had experience coming out as MSM. After PSM, the demographic characteristics analysis results showed homogeneity between the 2 groups.

**Table 2:** The comparison of the characteristics of men who have sex with men between 2 voluntary counseling and testing models.

Variable		Total (N=430)		Traditional VCT^a^ model (n=215)		Social networking VCT model (n=215)			*t* test (*df*)		Chi-square (*df*)		*P* value	
		Mean (SD)	n (%)	Mean (SD)	n (%)		Mean (SD)	n (%)						
Age (years)		29.97(7.61)	N/A^b^	30.03(7.8)	N/A		29.79(7.5)	N/A		−0.39 (214)		N/A		.70
**Education**									N/A		0.0 (2)		>.99	
	Above university	N/A	86 (20.0)	N/A	43 (20.0)		N/A	43 (20.0)						
	College or university	N/A	294 (68.4)	N/A	147 (68.4)		N/A	147 (68.4)						
	High school or less	N/A	50 (11.6)	N/A	25 (11.6)		N/A	25 (11.6)						
**Employment** **status**									N/A		0.0 (2)		>.99	
	Student	N/A	92 (21.4)	N/A	46 (21.4)		N/A	46 (21.4)						
	Employed	N/A	296 (68.8)	N/A	148 (68.8)		N/A	148 (68.8)						
	Unemployed	N/A	42 (9.8)	N/A	21 (9.8)		N/A	21 (9.8)						
**Religion**									N/A		0.2 (1)		.70	
	Yes	N/A	206 (47.9)	N/A	101 (47.0)		N/A	105 (48.8)						
	No	N/A	224 (52.1)	N/A	114 (53.0)		N/A	110 (51.2)						
**Coming out of sexual orientation**									N/A		0.97 (1)		.32	
	Yes	N/A	260 (60.5)	N/A	125 (58.1)		N/A	135 (62.8)						
	No	N/A	170 (39.5)	N/A	90 (41.9)		N/A	80 (37.2)						

^a^VCT: voluntary counseling and testing.

^b^N/A: not applicable.

### The Comparison of the Risk-Taking Behavior of MSM Between the 2 VCT Models

Compared with the traditional VCT model, some of the HIV risk-taking behaviors were significantly higher in the social networking VCT model (Table 3). The last unsafe sex time to date (in days) was 79.89 days shorter (t_209_=−12.16; *P*<.001) in the social networking VCT model than in the traditional VCT model. There were 9.8% (21/215; *χ*^2^_1_*=*5.7; *P*=.02) more participants in the social networking VCT model who had experience seeking sex through social media as compared with the traditional VCT model. The percentage of participants having multiple relationships with current sexual partners within the past 3 months in the social networking VCT model was 17.7% (38/215) more and significantly higher than the traditional VCT model (*χ*^2^_1_=14.5; *P*=.001). The mean number of anal intercourses within the past 3 months was 1.95 times significantly higher in the social networking VCT model than in the traditional VCT model (t_213_=3.2; *P*=.002). The social networking VCT model participants reported 10.2% (22/215) more of not using a condom every time and 3.3% (7/215) more of never used a condom during anal intercourse within the past 3 months, compared with the traditional VCT model participants (*χ*^2^_2_*=*8.8; *P*=.01). The experience of recreational drug use was 15.3% (33/215) more and was significantly higher among participants in the social networking VCT model than among those in the traditional VCT model (*χ*^2^_1_=16.6; *P*<.001). In total, 13.5% (29/215) more of the social networking VCT model participants reported having a history of a sexually transmitted disease, as compared with the traditional VCT model participants (*χ*^2^_1_*=*11.7; *P*=.001). There were 7.0% (15/215; *χ*^2^_1_*=*4.7; *P*=.03) and 8.8% (19/215; *χ*^2^_1_*=*7.1; *P*=.008) more of participants in the social networking VCT model who did not undergo HIV testing or regularly receive testing, respectively, than those in the traditional VCT model. There were no significant differences in the percentage of patients with PEP and PrEP between the two VCT models.

**Table 3:** The comparison of the risk-taking behavior of men who have sex with men between two voluntary counseling and testing models.

Variable		Total (N=430)		Traditional VCT^a^ model (n=215)		Social networking VCT model (n=215)		*t* test (*df*)	Chi-square (*df*)	*P* value
		Mean (SD)	n (%)	Mean (SD)	n (%)	Mean (SD)	n (%)			
Days since the last unsafe sex		65.2 (76.52)	N/A^b^	107.8 (84.71)	N/A	27.91 (37.59)	N/A	−12.2 (209)	N/A	<.001
**Seeking sexual activity through social media**								N/A	5.7 (1)	.02
	Yes	N/A	329 (76.5)	N/A	154 (71.6)	N/A	175 (81.4)			
	No	N/A	101 (23.5)	N/A	61 (28.4)	N/A	40 (18.6)			
**Relationship with the current sexual partner^c^**								N/A	14.5 (2)	.001
	Only one and stable	N/A	149 (34.7)	N/A	89 (41.1)	N/A	60 (27.9)			
	Multiple	N/A	180 (41.9)	N/A	71 (33.0)	N/A	109 (50.7)			
	Single	N/A	101 (23.5)	N/A	55 (25.6)	N/A	46 (21.4)			
Number of anal intercourses^c^		5.00 (6.83)	N/A	3.94 (4.24)	N/A	5.89 (8.39)	N/A	3.2 (213)	N/A	.002
**Condom use frequency during anal intercourse^c^**								N/A	8.8 (2)	.01
	Every time used	N/A	165 (38.4)	N/A	97 (45.1)	N/A	68 (31.6)			
	Not every time used	N/A	236 (54.9)	N/A	107 (49.8)	N/A	129 (60.0)			
	Never used	N/A	29 (6.7)	N/A	11 (5.1)	N/A	18 (8.4)			
**Experience of recreational drug use**								N/A	16.6 (1)	<.001
	Yes	N/A	81 (18.8)	N/A	24 (11.2)	N/A	57 (26.5)			
	No	N/A	349 (81.2)	N/A	191 (88.8)	N/A	158 (73.5)			
**History of sexual transmitted disease**								N/A	11.7 (1)	.001
	Yes	N/A	91 (21.2)	N/A	31 (14.4)	N/A	60 (27.9)			
	No	N/A	339 (78.8)	N/A	184 (85.6)	N/A	155 (72.1)			
**Having had a prior HIV test**								N/A	4.7 (1)	.03
	Yes	N/A	375 (87.2)	N/A	195 (90.7)	N/A	180 (83.7)			
	No	N/A	55 (12.8)	N/A	20 (9.3)	N/A	35 (16.3)			
**Having HIV test regularly (n=375)^d^**								N/A	7.1 (1)	.008
	Yes	N/A	268 (71.5)	N/A	151 (77.4)	N/A	117 (65.0)			
	No	N/A	107 (28.5)	N/A	44 (22.6)	N/A	63 (35.0)			
**Having PEP^e^** **previously**								N/A	2.7 (1)	.10
	Yes	N/A	33 (7.7)	N/A	12 (5.6)	N/A	21 (9.8)			
	No	N/A	397 (92.3)	N/A	203 (94.4)	N/A	194 (90.2)			
**Having PrEP^f^ previously**								N/A	1.8 (1)	.18
	Yes	N/A	40 (9.3)	N/A	16 (7.4)	N/A	24 (11.2)			
	No	N/A	390 (90.7)	N/A	199 (92.6)	N/A	191 (88.8)			

^a^VCT: voluntary counseling and testing.

^b^N/A: not applicable.

^c^Within the past 3 months.

^d^Within the past 1 year.

^e^PEP: postexposure prophylaxis.

^f^PrEP: pre-exposure prophylaxis.

### Comparison of the HIV Case Finding and Clinic Referrals of the 2 VCT Models

Table 4 compares HIV testing and referrals to the clinic results between the two models. The HIV positive rate was significantly higher in the social networking VCT model (13/215, 6.0%) than in the traditional VCT model (4/215, 1.9%; IRR 3.40, 95% CI 1.089-10.584; *P*=.03). The referral rate to the clinics for confirmation diagnosis and treatment after an HIV positive result was significantly higher in the social networking VCT model (12/13, 92%) than in the traditional VCT model (1/4, 25%) (IRR 0.03, 95% CI 0.001-0.585; *P*=.006).

**Table 4:** The comparison of the HIV case finding and clinic referrals between the 2 voluntary counseling and testing models.

Variable	Traditional VCT^a^ model (n=215), n (%)	Social networking VCT model (n=215), n (%)	IRR^b,c^	95% CI	*P* value
HIV positive	4 (1.9)	13 (6.0)	3.40	1.089-10.584	.03
Referred to the clinics for confirmation, diagnosis, and treatment	1 (25.0)	12 (92.3)	0.03	0.001-0.585	.006

^a^VCT: voluntary counseling and testing.

^b^IRR: incidence rate ratio.

^c^Incidence rate ratio was used to compare the difference of ratio.

## Discussion

### Principal Findings

This study applied mobile HIV testing via recruitment from social networking platforms and measured HIV case finding results. The two main findings were as follows: (1) compared with those receiving the traditional VCT model, the social networking VCT model of mobile HIV testing is more likely to reach MSM who have higher HIV risk-taking behaviors and (2) the HIV positive rates are 3 times significantly higher among those receiving the social networking VCT model than the traditional VCT model. There are three possible reasons why the HIV positive rate is higher in the social networking VCT model than in the traditional VCT model.

First, reloading into popular GSN apps in different locations could effectively enlarge the areas exposed to information about HIV testing to web-based MSM. As compared with the traditional VCT model, which provides public testing information on a certain website, the social networking VCT model was applied through the most popular GSN apps used by MSM [12,34,35]. Previous studies have indicated that MSM on social networking platforms also engage in more active unsafe sexual behaviors and do not regularly test for HIV [11-13,15-18,36]. The active presentation of heading and profiles in the social networking VCT model for nearby web-based users periodically and substantially increased mobile HIV testing information dissemination to high risk-taking MSM. Due to the delivery of VCT to the participant, the research assistant reloaded into GSN apps at an additional 55 locations, which increased the opportunity to reach broader web-based audiences [37] and avoided repeated recruitment [31]. In previous studies, pop-up and banner-paid advertisements and message sending were used to recruit MSM through GSN apps, and the range of the click-through rate was between 2.8% and 61.3% within 6 weeks to 4 months [12,22,38]. In our study, 4 times per day and a total of 1440 times of reloading into 3 GSN apps within 6 months without charge aroused 845 web-based users to click-tap, giving a click-through rate of 58.9%, which has the same effect of drawing attention as the paid advertisement. The reloading strategy of GSN apps with an attractive heading and profile of mobile HIV testing could successfully promote the attention and engagement of the research among nearby web-based MSM users, who had higher rates of HIV risk-taking behaviors.

Second, testing behavior was promoted through interactive and private message–based discussions of HIV risk-taking assessment through social networking platforms and flexible testing protocols. The HIV testing behavior of MSM could be motivated through the interactive and stimulating web-based counseling about risk-taking awareness and testing resources [23,39,40] and offering a flexible time and location for HIV screening to meet the participants’ needs [41]. In our study, the average completion rate of the social networking VCT model after a one-on-one discussion with web-based users was 20.8%, which is higher than that of the traditional VCT model (8.56%). The client’s own house was the most preferred place for the participants in the social networking VCT model because of privacy and convenience [42]. Although no injurious event was reported while providing the mobile HIV testing at clients’ homes, it is worth noting that safety procedures were in place before going to the designated location. These included informing the team member of the time and place, setting up an emergency call button on the home page of the cell phone, and, if there were possible dangers during the test, arranging for a colleague to accompany the service provider as necessary. Compared with previous studies that applied the mobile HIV testing in a specific shelter and trunk [8-10], this study found that the mobile HIV testing could be conducted in a daily life environment, such as the seating area of convenience stores and restaurants around an MRT station in Taiwan.

Third, referrals were made via the risk-taking sexual network by those participants who had completed the mobile HIV testing of the social networking VCT model through the convenience of the Line app. MSM undergoing HIV testing usually self-identify as being exposed to risk-taking sexual behaviors and/or many sexual partners [43,44]. The acquisition of HIV testing information through network-mediated MSM could significantly predict more HIV testing behaviors than other models [45]. In addition to encouraging the sharing of HIV testing information to the network of those who had finished the social networking VCT model, we also provided a convenient referral link by using the quick response code of the Line app. Referral of those MSM with HIV risk-taking could be facilitated while applying the information linking function and immediate web-based discussion within the Line app.

Higher referral rates to the clinic after testing HIV positive in the social networking VCT model than the traditional VCT model may be attributed to the fact that the social networking VCT model could be arranged during the daytime, providing enough time for the research assistant to accompany the positive participants immediately to the hospital for a confirmation test and further treatment. Moreover, 6.0% (12/199) of the HIV-negative participants in the social networking VCT model were referred to the PEP or PrEP clinic by our research assistant, which is higher than the traditional VCT model that had no referrals to the PEP or PrEP. The flexible HIV testing algorithms increased the chance of accessing treatment after an HIV-positive result and being given information regarding preventive measures after HIV-negative results [46].

### Limitations

This study has several limitations that need to be addressed. First, the sources of the recruited participants were mainly Taipei and New Taipei City, which is a metropolitan area with convenient transportation that may limit the generalizability and extension of the results to rural areas. Second, self-report questionnaires may be skewed toward social desirability and could influence the validity of the results. Third, the implementation methods differed in several aspects between the two VCT models. These included client mobilization, recruitment methods, and testing schedules. Therefore, the effects of the social networking VCT model on HIV case findings are unclear. Fourth, the design and methods of this study require well-trained full-time personnel. Therefore, it is difficult to perform the social networking VCT model in a real-world setting when human resources and budgets are in shortage.

### Conclusions

Compared with the traditional VCT model, the social networking VCT model could successfully recruit web-based MSM with a higher risk-taking of HIV by periodically reloading into social networking platforms and having discussions. The VCT is delivered in flexible testing times and locations, which increases the motivation for HIV testing behavior. Referrals to the clinic for the confirmation of diagnosis and treatment, and for PEP or PrEP, are also feasible after the social networking VCT model. The cost-effectiveness and more rigorous design of the social networking VCT model could be assessed in the future to evaluate the outcomes and increase clinical receptivity.

## Abbreviations

FB: Facebook
GSN: geosocial network
IRR: incidence rate ratio
MRT: mass rapid transportation
MSM: men who have sex with men
PEP: postexposure prophylaxis
PrEP: pre-exposure prophylaxis
PSM: propensity score matching
VCT: voluntary counseling and testing
